# Assay dependence of *Brucella* antibody prevalence in a declining Alaskan harbor seal (*Phoca vitulina*) population

**DOI:** 10.1186/1751-0147-55-2

**Published:** 2013-01-16

**Authors:** Karsten Hueffer, Scott M Gende, Todd M O’Hara

**Affiliations:** 1 Institute of Arctic Biology and Department of Biology and Wildlife, University of Alaska Fairbanks, 902 N. Koyukuk Dr., Fairbanks, AK 99775, USA; 2 National Park Service, Glacier Bay Field Station, 3100 National Park Road, Juneau, Alaska 99801, USA

**Keywords:** *Brucella*, Harbor seals, Alaska, Assay dependence

## Abstract

**Background:**

*Brucella* is a group of bacteria that causes brucellosis, which can affect population health and reproductive success in many marine mammals. We investigated the serological prevalence of antibodies against *Brucella* bacteria in a declining harbor seal population in Glacier Bay National Park, Alaska.

**Results:**

Prevalence ranged from 16 to 74 percent for those tests detecting antibodies, indicating that harbor seals in Glacier Bay have been exposed to *Brucella* bacteria. However, the actual level of serological prevalence could not be determined because results were strongly assay-dependent.

**Conclusions:**

This study reinforces the need to carefully consider assay choice when comparing different studies on the prevalence of anti–*Brucella* antibodies in pinnipeds and further highlights the need for species- or taxon-specific assay validation for both pathogen and host species.

## Background

Harbor seal (*Phoca vitulina*) populations serve many important biological and sociocultural functions in Alaska. Harbor seals are apex predators and serve as an important prey for species such as killer whales (*Orcinus orca*) and Steller sea lions (*Eumetopias jubatus*) [1-3]. Harbor seals are also important subsistence food for many local communities, and play a prominent role in the culture of the Huna-Tlingit and other tribes. Glacier Bay National Park (GBNP) supports a unique stock of harbor seals in Alaska [4] and historically was the location of the largest breeding population of seals in the state. Yet the population has been declining since 1992 [5] even though foraging and other conditions have supported significant increases in other marine mammals, such as Steller sea lions and humpback whales, that utilize the park during the summer [6,7]. Hypotheses for the causal factors driving the decline include anthropogenic disturbance, although a recent study demonstrated that disturbance is not likely to be sufficiently frequent to influence population dynamics [8]. Large populations of forage fish persist in GBNP [9], making food availability unlikely as a possible explanation for the population decline, and seals that breed in Glacier Bay are of similar physiological condition as those in other stable or increasing populations [10]. Predation by the increasing population of Steller sea lions has also been proposed although this is not likely to be the sole factor driving population dynamics [3]. Together then a number of hypotheses (food availability, disturbance, predation) have been addressed with no significant evidence identifying the cause for the observed decline in this population.

Here we perform a serological survey for exposure to *Brucella* bacteria, pathogens of concern in marine mammals in Alaska. In a recent study we determined the prevalence of antibodies against *Leptospira* spp., *Toxoplasma gondii* and distemper viruses as well as presence of *Giardia* and *Cryptosporidium* in fecal samples from this population [11] but we did not investigate the presence of *Brucella* bacteria, which are known to be present in harbor seals in Southeast Alaska [12]. *Brucella* bacteria have been identified as pathogens in marine mammals since 1994 [13] and have since been isolated, or anti–*Brucella* antibodies have been detected, in multiple marine mammal species throughout the world [14,15]. Brucellosis is a bacterial infection that can affect reproductive organs and therefore influence fecundity and lead to reduced recruitment and alter population dynamics [15], although in pinnipeds overt pathological findings have so far not been observed [14]. In addition, marine-derived *Brucella* bacteria have significant zoonotic potential in people exposed to marine mammals [16-18].

In this study we tested serum samples described previously [11] for anti–*Brucella* antibodies using six different tests in order to gain insight into the serological prevalence to assess the possible exposure to *Brucella* bacteria in Glacier Bay harbor seals.

## Methods

### Samples

In order to determine if the harbor seal population in GBNP had been exposed to *Brucella* bacteria we performed a number of serological tests to detect anti–*Brucella* antibodies in seals captured in 2007 (49 animals) and 2008 (44 animals) from harbor seals in Johns Hopkins Inlet (58.84N 137.11W), Glacier Bay National Park (GBNP), Alaska as described previously [11]. Age classes were determined [19], and 46 animals were classified as pups, 19 as yearlings, 10 as subadults, and 18 as adults. 51 animals were female and 42 were male.

All animal sampling was in accordance with approval of Institutional Animal Care and Use Committees at the University of Alaska Fairbanks (protocol 07–37) and the State of Alaska Department of Fish and Game (protocol 07–16), as well as a permit from the National Oceanographic and Atmospheric Administration under the Marine Mammal Protection Act (Permit 358-1787-00).

### Serological tests

#### Brucellosis card test

Brucellosis Card tests (Becton Dickinson, Cockeysville, MD, US) using *B. abortus* strain 1119–3 as the antigen was performed independently according to manufacturer’s instructions at the University of Alaska Fairbanks and the diagnostic laboratory of Colorado State University to ensure consistency between operators.

#### *B. abortus* plate test

Harbor seal serum was pipetted onto etched glass plates. Standard *B. abortus* Standard Plate Antigen (Strain 1119–3, National Veterinary Services Laboratories, Ames, Iowa, US) was added and thoroughly mixed with the serum and the plate rotated and incubated for 8 minutes further rotated before agglutination was assessed in indirect light over a black background.

#### Competitive ELISA

Competitive ELISA was performed at Mystic Aquarium. This test uses an antigen derived from *Brucella* isolated from a harbor seal. Serum samples were tested at a 1:10 dilution and less than 25% inhibition was considered negative. 25–29.9% inhibition was classified as a suspect test and sera showing 30% or higher inhibition were classified as positive for antibodies to “marine” *Brucella* spp. [20].

#### *B. ovis* ELISA as well as *B. canis* RSAT

*B. ovis* ELISA as well as *B. canis* RSAT were performed at the diagnostic laboratory of Colorado State University. The *B. ovis* ELISA followed the NVSL SeroPro protocol using the REO198 Antigen. The *B. canis* RSAT test utilized a commercially available test kit (D-TEC®, Synbiotics, Kansas City, MO)**.**

### Statistical analysis

The 95% confidence intervals for serological prevalence were calculated as previously described [21]. The different tests were compared by calculating positive percentage agreement, negative percent agreement and the overall percent agreement as well as McNemar’s chi square test for pair-wise comparison of the diagnostic assays used in this study.

## Results

**Figure 1:**
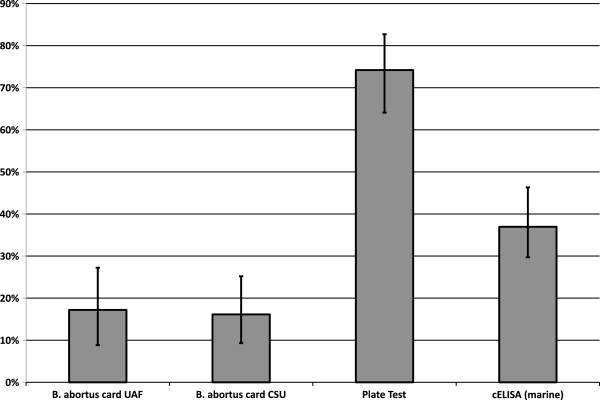
**Serological prevalence of anti–*Brucella *antibodies for different tests.** The percentage of positive reaction is shown with a 95% upper and lower confidence interval. Assays for antibodies towards *B. ovis* and *B. canis* did not detect antibodies in any samples (data not presented).

Using an ELISA assay detecting *B. ovis* antibodies and a rapid slide agglutination test (RSAT) detecting *B. canis* antibodies, we did not detect an antibody positive sample in 93 animals tested. A Plate test for anti–*Brucella* antibodies yielded a 74% (95% CI = 64-82%) serological prevalence rate. The commercially available card test used detected antibodies against *B. abortus* in 17% (95% CI = 10-26%) and 16% (95% CI = 9-25%) samples for UAF and CSU, respectively. To confirm these results we performed this test independently and obtained very similar results with 95% overall agreement. The competitive ELISA based on a marine *Brucella* isolate detected antibodies in 37% (95% CI = 27-47%) (Figure 1).

Using a McNemar’s Chi square test, the correlation between the different assays showed great differences between results with the plate test having the greatest rate of positive reactions. All tests for anti–*Brucella* antibody showed significant differences (*P* < 0.001) in pair-wise comparisons, except the card test done at CSU compared to the card test at UAF (Table 1).

**Table 1:** Pair-wise comparison of results from different serological tests


A		UAF card neg	UAF card pos	Total
	cELISA neg	57	1	58
	cELISA pos	19	15	34
	Total	76	16	92
		NPA: 75	PPA: 93	TPA: 78
	McNemar’s chi^2^: 14.45		p < 0.001	
B		UAF card neg	UAF card pos	Total
	plate neg	24	0	24
	plate pos	53	16	69
	Total	77	16	93
		NPA: 31	PPA: 100	TPA: 43
	McNemar’s chi^2^: 51.01		p < 0.001	
C		UAF card neg	UAF card pos	Total
	CSU card neg	75	3	78
	CSU card pos	2	13	15
	Total	77	16	93
		NPA: 97	PPA: 81	TPA: 95
	McNemar’s chi^2^: 0		p > 0.1	
D		plate neg	plate pos	Total
	cELISA neg	20	38	58
	cELISA pos	4	30	34
	Total	24	68	92
		NPA: 83	PPA: 44	TPA: 54
	McNemar’s chi^2^: 25.93		p < 0.001	
E		plate neg	plate pos	Total
	CSU card neg	23	55	78
	CSU card pos	1	14	15
	Total	24	69	93
		NPA: 96	PPA: 20	TPA: 40
	McNemar’s chi^2^: 50.16		p < 0.001	
F		cELISA neg	cELISA pos	Total
	CSU card neg	55	22	77
	CSU card pos	3	12	15
	Total	58	34	92
		NPA: 95	PPA: 35	TPA: 73
	McNemar’s chi^2^: 12.96		p < 0.001	

The results for two tests were compared for all samples to determine relationship between results of different tests. Test agreement is represented as negative (NPA), positive (PPA) and total (TPA) predictive agreement. Results for the McNemar’s Chi^2^ test are represented as Chi^2^ and the corresponding p value for 1 df. Assays for *B. ovis* and *B. canis* results are not included here.

## Discussion

This study is an extension of a previous assessment of exposure of infectious agents in this declining harbor seal population of GBNP [11], our work on brucellosis in polar bears [22] and moose (*Alces alces) *[23,24], and of other investigators working on Alaska mammals [12,25]. In the previous GBNP study that used the same serum samples as the present study, antibodies to *Leptospira* ssp. were detected while anti-distemper antibodies were not detected, potentially leaving the population in Glacier Bay vulnerable to this severe viral disease [11]. To assess if exposure to *Brucella* bacteria was evident and could be involved in the past and current population decline in the Glacier Bay harbor seal population, we tested serum from harbor seals from this area for the presence of anti–*Brucella* antibodies. Based on the uncertainty of *Brucella* species present in pinnipeds in the North Pacific as well as assay dependent, cross reactivity with other bacterial pathogens [26] we tested the sera using six different assays. Serological prevalence rates differed between assays detecting antibodies over a range of 16 to 74 percent of samples. Two assays failed to detect antibody, which was expected as *B. ovis* and *B. canis* have not been described in harbor seals previously and would likely not cross react with *Brucella* organism(s) known to be circulating in harbor seal populations as they have rough lipopolysaccharides (LPS), indicating that the harbor seal most likely have been exposed to *Brucella* species exhibiting a smooth phenotype. These results indicate that *Brucella* bacteria are present in the harbor seal population of Glacier Bay; however the actual serological prevalence rate cannot be determined as different assays gave widely varying results. Prevalence of anti–*Brucella* antibodies ranged from 0 to 81 percent in other studies [20,27-31] with variation attributed to annual differences [30] and assay type [29]. Together, these findings highlight the need for cautious interpretation of serological results of exposure to *Brucella* bacteria.

Comparing the results from diagnostic tests without knowing the true prevalence of positive samples is difficult. No validated gold standard exists for the detection of antibodies against *Brucella* in pinnipeds, and developing such a standard for all marine mammal species and *Brucella* species is logistically difficult and cost prohibitive [27]**.** Using total predictive agreement as a measure, the plate test shows less than 55% agreement with the other tests. The card test and the cELISA agreed reasonably well, with over 70% total predictive agreement (Table 1). While this agreement is useful at a population level, it is less useful for assessing the status of individual animals. The apparent higher serological prevalence using the plate test could be explained by the conversion of fibrinogen to fibrin, which can lead to false positive results [27]; cross reactivity with antibodies to other gram negative bacteria could be an alternative explanation. However, the card test employed the same antigen and did not result in such high prevalence rates, indicating that cross reactivity of antibodies against other pathogens with the antigen used in this assay is not the main reason for the higher rate of positive samples when using the plate test.

Due to the assay dependence of serological prevalence and the uncertain pathology of brucellosis in pinnipeds, the influence of brucellosis on the harbor seal population in GBNP cannot be established. Because the serological tests used in this study are designed to detect past exposure, no conclusions can be drawn on current infection status or if *Brucella* bacteria are causing adverse health effects. However, a potential role of Brucellosis in the possible multifactorial population decline cannot be excluded. In addition our results show the presence of a zoonotic agent in this population, which should be taken into consideration in any management and animal handling decisions.

The assay dependent differences in apparent exposure rates to *Brucella* bacteria reinforce the need for a careful approach to comparing literature on exposure to *Brucella* bacteria in pinnipeds using common diagnostic approaches, which are often not developed for or validated in marine mammals. While biological factors, such as age of animals sampled [12] are important in comparing different studies on *Brucella* exposure prevalence in pinnipeds, the serological test used should be considered an additional and possibly the main source of variation. Studies can only be compared and used to determine trends if similar assays were used in previous studies employing similar methods (including criteria to deem a sample positive). As most commonly used tests for antibodies against *Brucella* are based on methodology developed for livestock, companion animals, or humans, the marine cELISA used in this study is likely the most appropriate among the tests used here. In addition this test has been validated in detecting antibodies in harbor seals with brucellosis. It’s also a competitive ELISA and thus increases the usefulness across different host taxa as reactivity to antibodies of the host by secondary antibodies is not necessary. However the possibility that different biovars exist between *B. pinnipedia* and uncertainty about *Brucella* spp. distribution in marine mammals should lead to careful interpretation of even this assay, which is based on a marine derived *Brucella* isolate. We strongly suggest that if results from a study are intended to be compared to previously conducted serosurveys in pinnipeds that the same test from the historic study be included alongside the marine based cELISA.

## Conclusions

In this study we show that the declining harbor seal population in GBNP has been exposed to *Brucella* bacteria and that these pathogens could play a role in affecting the health of this population. Prevalence of antibodies against *Brucella* bacteria varied greatly between assays. Based on validation of this assay in harbor seals described in the literature the marine based cELISA is identified as the most appropriate test employed here.

## Competing interests

The authors declare no competing interests.

## Authors’ contributions

TMO and SMG conceived and performed the study. KH analyzed the data and wrote the manuscript. All authors read and approved the final manuscript.

## References

[B1] Ford JKB Ellis GM Barrett-Lennard LG Morton AB Palm R Balcomb KC Dietery specialization in two sympatric populations of killer whales (Orcinus orca) in coastal British Columbia and adjacent waters Can J Zool 1998 76 1456 1471

[B2] Herreman JK Blundell GM Ben-David M Evidence of bottom-up control of diet driven by top-down processes in a declining harboer seal Phoca vitulina richardsi population Mar Ecol Prog Ser 2009 374 287 300

[B3] Mathews EA Adkison MD The role of Steller sea lions in a large population decline of harbor seals Marine Mammal Science 2010 26 803 836 10.1111/j.1748-7692.2010.00375.x

[B4] Allen BM Abngliss RP Alaska Marine Mammal Stock Assessments: Harbor seal National Marine Fisheries Services NOAA-TM-AFSC-234 2011

[B5] Womble JN Pendleton GW Mathews EA Blundell GM Bool NM Gende SM Harbor seal (Phoca vitulina richardii) decline continues in the rapidly changing landscape of Glacier Bay National Park, Alaska 1992–2008 Marine Mammal Science 2010 26 686 697

[B6] Mathews EA Womble JN Pendleton GW Maniscalco JM Streveler G Population growth and colonization of Steller sea lions in the Glacier Bay region of southeastern Alaska:1970s–2009 Marine Mammal Science 2011 27 852 880 10.1111/j.1748-7692.2010.00455.x

[B7] Hendrix AN Straley J Gabriele CM SM G Bayesian estimation of humpback whale (Megaptera novaeangliae) population abundance and movement patterns in southeastern Alaska Can J Fish Aquat Sci 2012 69 1 15 10.1139/f2011-128

[B8] Young C Disturbance of harbor seals by vessels in Johns Hopkins Inlet, Glacier Bay 2009 AK. MS thesis: San Jose State University

[B9] Arimitsu ML Piatt JF Litzow MA Abookire AA Romano MD Robards MD Distribution and spawning dynamics of capelin (Mallotus villosus) in Glacier Bay, Alaska: a cold water refugium Fish Oceanogr 2008 17 137 146 10.1111/j.1365-2419.2008.00470.x

[B10] Blundell GM Womble JN Pendleton GW Karpovich SA Gende SM Herreman JK Use of glacial and terrestrial habitats by harbor seals in Glacier Bay, Alaska: costs and benefits Mar Ecol Prog Ser 2011 429 277 290

[B11] Hueffer K Holcomb D Ballweber LR Gende SM Blundell G O’Hara TM Serologic surveillance of pathogens in a declining harbor seal (Phoca vitulina) population in Glacier Bay National Park, Alaska, USA and a reference site J Wildl Dis 2011 47 984 988 22102671 10.7589/0090-3558-47.4.984

[B12] Zarnke RL Saliki JT Macmillan AP Brew SD Dawson CE Ver Hoef JM Frost KJ Small RJ Serologic survey for Brucella spp., phocid herpesvirus-1, phocid herpesvirus-2, and phocine distemper virus in harbor seals from Alaska, 1976–1999 J Wildl Dis 2006 42 290 300 16870851 10.7589/0090-3558-42.2.290

[B13] Ross HM Foster G Reid RJ Jahans KL MacMillan AP Brucella species infection in sea-mammals Vet Rec 1994 134 359 8017020 10.1136/vr.134.14.359-b

[B14] Nymo IH Tryland M Godfroid J A review of Brucella infection in marine mammals, with special emphasis on Brucella pinnipedialis in the hooded seal (Cystophora cristata) Vet Res 2011 42 93 10.1186/1297-9716-42-93 21819589 PMC3161862

[B15] Foster G MacMillan AP Godfroid J Howie F Ross HM Cloeckaert A Reid RJ Brew S Patterson IA A review of Brucella sp. infection of sea mammals with particular emphasis on isolates from Scotland Vet Microbiol 2002 90 563 580 10.1016/S0378-1135(02)00236-5 12414172

[B16] McDonald WL Jamaludin R Mackereth G Hansen M Humphrey S Short P Taylor T Swingler J Dawson CE Whatmore AM Stubberfield E Perrett LL Simmons G Characterization of a Brucella sp. strain as a marine-mammal type despite isolation from a patient with spinal osteomyelitis in New Zealand J Clin Microbiol 2006 44 4363 4370 10.1128/JCM.00680-06 17035490 PMC1698438

[B17] Sohn AH Probert WS Glaser CA Gupta N Bollen AW Wong JD Grace EM McDonald WC Human neurobrucellosis with intracerebral granuloma caused by a marine mammal Brucella spp Emerg Infect Dis 2003 9 485 488 10.3201/eid0904.020576 12702232 PMC2957978

[B18] Whatmore AM Dawson CE Groussaud P Koylass MS King AC Shankster SJ Sohn AH Probert WS McDonald WL Marine mammal Brucella genotype associated with zoonotic infection Emerg Infect Dis 2008 14 517 518 10.3201/eid1403.070829 18325282 PMC2570841

[B19] Blundell GM Pendleton GW Estimating age of harbor seals (Phoca vitulina) with incisor teeth and morphometrics Marine Mammal Science 2008 24 577 590 10.1111/j.1748-7692.2008.00194.x

[B20] Meegan J Field C Sidor I Romano T Casinghino S Smith CR Kashinsky L Fair PA Bossart G Wells R Dunn JL Development, validation, and utilization of a competitive enzyme-linked immunosorbent assay for the detection of antibodies against Brucella species in marine mammals J Vet Diagn Invest 2010 22 856 862 10.1177/104063871002200603 21088168

[B21] Hughes-Hanks JM Rickard LG Panuska C Saucier JR O’Hara TM Dehn L Rolland RM Prevalence of Cryptosporidium spp. and Giardia spp. in five marine mammal species J Parasitol 2005 91 1225 1228 10.1645/GE-545R.1 16419775

[B22] O’Hara TM Holcomb D Elzer P Estepp J Perry Q Hagius S Kirk C Brucella species survey in polar bears (ursus maritimus) of northern Alaska J Wildl Dis 2010 46 687 694 20688674 10.7589/0090-3558-46.3.687

[B23] O’Hara TM Dau J Carroll G Bevins J Zarnke R Evidence of exposure to Brucella suis biovar 4 in northern Alaska moose Alces 1998 34 31 40

[B24] Edmonds MD Ward FM O’Hara TM Elzer PH Use of western immunoblot analysis for testing moose serum for Brucella suis biovar 4 specific antibodies J Wildl Dis 1999 35 591 595 10479098 10.7589/0090-3558-35.3.591

[B25] Burek KA Gulland FM Sheffield G Beckmen KB Keyes E Spraker TR Smith AW Skilling DE Evermann JF Stott JL Saliki JT Trites AW Infectious disease and the decline of Steller sea lions (Eumetopias jubatus) in Alaska, USA: insights from serologic data J Wildl Dis 2005 41 512 524 16244061 10.7589/0090-3558-41.3.512

[B26] Godfroid J Nielsen K Saegerman C Diagnosis of brucellosis in livestock and wildlife Croat Med J 2010 51 296 305 10.3325/cmj.2010.51.296 20718082 PMC2931434

[B27] Nielsen O Nielsen K Braun R Kelly L A comparison of four serologic assays in screening for Brucella exposure in Hawaiian monk seals J Wildl Dis 2005 41 126 133 15827218 10.7589/0090-3558-41.1.126

[B28] Tryland M Sorensen KK Godfroid J Prevalence of Brucella pinnipediae in healthy hooded seals (Cystophora cristata) from the North Atlantic Ocean and ringed seals (Phoca hispida) from Svalbard Vet Microbiol 2005 105 103 111 10.1016/j.vetmic.2004.11.001 15627521

[B29] Aguirre AA Keefe TJ Reif JS Kashinsky L Yochem PK Saliki JT Stott JL Goldstein T Dubey JP Braun R Antonelis G Infectious disease monitoring of the endangered Hawaiian monk seal J Wildl Dis 2007 43 229 241 17495307 10.7589/0090-3558-43.2.229

[B30] Lynch M Duignan PJ Taylor T Nielsen O Kirkwood R Gibbens J Arnould JP Epizootiology of Brucella infection in Australian fur seals J Wildl Dis 2011 47 352 363 21441188 10.7589/0090-3558-47.2.352

[B31] Tryland M Nymo IH Nielsen O Nordoy ES Kovacs KM Krafft BA Thoresen SI Asbakk K Osterrieder K Roth SJ Lydersen C Godfroid J Blix AS Serum chemistry and antibodies against pathogens in antarctic fur seals, Weddell seals, crabeater seals, and Ross seals J Wildl Dis 2012 48 632 645 22740529 10.7589/0090-3558-48.3.632

